# Influence of intra and inter species variation in chilies (*Capsicum* spp.) on metabolite composition of three fruit segments

**DOI:** 10.1038/s41598-021-84458-5

**Published:** 2021-03-02

**Authors:** Tilen Zamljen, Jerneja Jakopič, Metka Hudina, Robert Veberič, Ana Slatnar

**Affiliations:** Department of Agronomy, Biotechnical Faculty, University of Ljubljana, Jamnikarjeva 101, 1000 Ljubljana, Slovenia

**Keywords:** Natural variation in plants, Secondary metabolism

## Abstract

Twenty-one different cultivars from four different species were examined. The highest dry weight was present in seeds (between 35 and 50%) and the average water content was 60%. Placenta and pericarp contained on average 86% water. Total sugars variation between species was 60%. The most concentrated in the various cultivar pericarps were ascorbic acid ranging from 368.1 to 2105.6 mg/100 g DW and citric acid ranging from 1464.3 to 9479.9 mg/100 g DW. Total phenolic content ranged from 2599.1 mg/100 DW in ‘Chilli AS- Rot’ to 7766.7 mg/100 g DW in 'Carolina Reaper'. The placenta had 23.5 times higher phenolic content than seeds. *C. chinense* and *C. chinense* × *C. frutescens* had 3.5 to 5 times higher capsaicinoid content compared to *C. annuum* and *C. baccatum*, with 'Carolina Reaper' having the highest content at 7334.3 mg/100 g DW and 'Chilli AS- Rot' the lowest (318.7 mg/100 g DW).

## Introduction

Chilies are plants belonging to the genus *Capsicum* (Solanaceae)^1^. *Capsicum annuum* L. is the most commonly cultivated species^2^. It includes pungent and non-pungent or sweet cultivars. It is also the economically most important of all of the *Capsicum* species. For *C. annuum* species, the pungency values range from 0 SHU (Scoville heat units) to 100,000 SHU. The fruits of *Capsicum baccatum* L. have different shapes and sizes, which is a common feature of this species, since other species have more uniform and comparable fruits^3^. The pungency levels in *C. baccatum* are between 30,000 SHU and 50,000 SHU^3^. The hottest chili cultivars can be found in the species *Capsicum chinense* Jacq^4^. High levels of pungency are a characteristic of this species. Fruits usually have a thin pericarp and a very large amount of placental tissue. The sharpness range extends from 100,000 to 2,000,000 SHU units^5^.

A chili fruit consists of three parts: pericarp, placenta and seeds. The outer skin and the pericarp usually have a cultivar specific color, which can vary from white, yellow tones to orange, red or even purple and brown. Some species have thicker and others have thinner pericarp walls^2^. The placenta containing the seeds is white, yellow, orange, red or green. It is usually similar in color to the pericarp. Placental tissue attaches itself to the walls of the carpel, so that the inside of the fruit is segmented into three to four cavities. The placenta can extend to the tip of the fruit. Bubbles filled with capsaicinoid-rich oils form in the placental tissue and it is the main site of capsaicinoid synthesis^6^.

The 5 most common capsaicinoids in hot peppers are capsaicin, dihydrocapsaicin, nordihydrocapsaicin, homocapsaicin and homodihydrocapsaicin. Capsaicin and dihydrocapsaicin account for 85—95% of all capsaicinoids in hot peppers^1^. They are also the sharpest, with 16 million Scoville heat units (SHU) on the Scoville scale in their pure crystal state^7^. Peppers are also a good source of organic acids, such as ascorbic acid (vitamin C), and sugars, especially red peppers. The largest quantities of seeds are found at the top of the chili. Further down in the fruit, the number of seeds decreases, so that there are usually no seeds at the tip^5^.

Because of their high nutritional value and their positive effects on human health, studies are focusing on how to increase and optimize the synthesis of certain metabolites. Various horticultural, chemical and physical approaches can be used to determine which species and cultivars are optimal for mass production^8^ and which parts of the fruit are most beneficial to human health. The aim of this study was to evaluate how the species and cultivar of chili affects the overall primary and secondary metabolite composition of different parts of the fruit, and not only certain metabolites. Majority of cultivars in our study, belonging to different species, were analyzed for primary and secondary metabolites for the first time. Also reported for the first time were twenty-one fruit segments and cultivar-specific capsaicinoid profiles. They include the determination of capsaicin, dihydrocapsaicin and nordihydrocapsaicin, as well as homocapsaicin and homodihydrocapsaicin, both of which were mostly left out in previous studies due to their low concentrations. Our study reveals sugar and organic acid profiles that are immensely important for the human taste perception of chilies. Chilies are sensitive to environmental factors^9^, which contributes to changes in metabolic composition. These changes have been reported in many studies^4,5,9,10^. All plants in this study were exposed to the same environmental and agricultural factors, which negates the influence of these factors and increases the quality of this study. The new findings can be used to further investigate new methods to increase metabolite production in chilies, with which the market price in some sectors is determined by the quality of the fruit rather than the quantity^11^.

## Materials and methods

Twenty-one different cultivars of chili plants were planted, 12 of which belonged to the *C. chinense* species, 5 belonged to the *C. annuum* species, 3 belonged to the *C. baccatum* species and one was a cross between *C. chinense* × *C. frutescens*. All plants were grown in pots in a plastic greenhouse from 22th May 2019 to 10th October 2019. Each pot was irrigated with the same amount of irrigation water via a drip irrigation system. At least three fruits were collected from each plant, with three plants of each variety being collected per treatment when they reached the variety-specific color. Whole fruits, placenta, seeds and pericarp were weighed before freeze-drying. The samples were lyophilized (freeze-dried) in a lyophilizer. After freeze-drying, the samples were weighed again so that we could calculate the dry weight of the samples. After weighing, the samples of pericarp, placenta and seeds were crushed in a cooled mortar until a fine powder was formed. For each cultivar, three repetitions, consisting of one to three fruits were performed for extraction and analysis.

### Extractions of sugars and organic acids

For extraction of sugars and organic acids, 0.05 g of powder was weighed and poured over with 2 ml of bidistilled water. Only edible parts (pericarp and placenta) were used for total sugars and organic acids analysis. Samples were then shaken on an orbital shaker for 30 min. After shaking, they were placed in a cooled centrifuge in which the samples were rotated at 10,000 rpm for 8 min. Samples were filtered through 25 µl cellulose filters (Chromafil A-25/25; Macherey–Nagel, Düren, Germany) and saved at – 20 °C until analysis on the Thermo Finnigan Surveyor HPLC system (Thermo Scientific, San Jose, USA). Two columns were used for the analysis: for sugars (Rezex RCM-monosaccharide (30 × 0.78 cm; Thermo Scientific, San Jose, USA)) and for organic acids (Rezex ROA organic acid column (30 × 0.78 cm; Phenomenex, Torrance, USA). Extraction method and HPLC settings were based on Zamljen et al.^12^ The results are reported in g/kg dry weight (DW) for sugars, citric, malic and quinic acid and in mg/100 g DW for succinic, fumaric and oxalic acid. All sugars and organic acids (including ascorbic acid) were determined only in edible parts (pericarp and placenta).

### Extractions of ascorbic acid

For extraction of ascorbic acid, 0.05 g of dry pericarp and 0.02 g of dry placenta were used. Pericarp samples were extracted using 4 ml (2 ml placental tissue) of bidistilled water containing 2% metaphosphoric acid. Similar as for the extraction of sugars and organic acids, ascorbic acid samples were shaken on an orbital shaker for 30 min and then centrifuged and filtered through a cellulose filter (Chromafil A-25/25; Macherey–Nagel, Düren, Germany). The HPLC settings and column were same as the analysis of the other organic acids. The only difference was that the column was cooled for ascorbic acid analysis. The results are given in mg/100 g DW.

### Extraction of phenolic and capsaicnoids

From each sample, 0.05 g of powder was weighed into a plastic tube. Total phenolics and capsaicinoids were extracted with 80% methanol. All samples were then placed in a cooled ultrasonic bath (0 °C) for 1 h. After the ultrasonic bath, the samples were centrifuged at 8000 rpm for 6 min and filtered through a 25 µl polyamide filter (Chromafil AO-45/25, Macherey–Nagel, Düren, Germany).

### Determination of total phenolic content

The total phenolic content (TPC) of the samples was determined using the Folin-Ciocalteu phenol reagent method^13^. For the analysis, 100 μl of sample, 500 μl of FC, 1.5 ml 20% Na_2_CO_3_ and 7.9 ml of bidistilled water was used. Prepared samples were placed in an oven preheated to 40 °C, for 30 min. Absorbance at 765 nm was measured using a Lambda Bio 20 UV/vis spectrophotometer (Perkin Elmer, Waltham, MA). TPC was determined as gallic acid equivalents (GAE) in mg/100 g of dry weight (DW).

### Determination of capsaicinoids

The same extract used for TPC was also used for the analysis of individual capsaicinoids. We analyzed five different capsaicinoids (capsaicin, dihydrocapsaicin, nordihydrocapsaicin, homocapsaicin and homodihydrocapsaicin) in our experiment, using a UHPLC – PDA Thermo Scientific Dionex UltiMate 3000 HPLC (Thermo Scientific) system, combined with a TSQ Quantum Access Max quadrupole mass spectrometer (MS) (Thermo Fischer Scientific Institute, Waltham, MA, USA). Capsaicinoid separation was done with a Gemini C18 (Phenomenex) column operating at 25 °C. The mobile phases were 0.1% formic acid and 3% acetonitrile in bidistilled water (A) and 0.1% formic acid and 3% bidistilled water in acetonitrile (B). Wang et al.^14^ gradient method was used. The flow rate was set to 0.6 ml/min and the injection volume of the sample was 20 μl. A TSQ Quantum Access Max quadrupole mass spectrometer (MS) detector was used for analysis. The presence of capsaicinoids was confirmed on a TSQ Quantum Access Max quadrupole mass spectrometer by Thermo Scientific, applying the following parameters: (ESI) source in positive ion mode operating at 300 °C, corona voltage 4.5 kV, sheath gas 90 l/h, auxiliary gas 60 l/h; mass spectra scanned in the range from *m*/*z* 90 to 700. Collision‐induced dissociation was achieved using argon as the collision gas in the collision cell. Capsaicinoids were analyzed in selected reaction monitoring (SRM) mode: for capsaicin *m/z* 306.2 > 137.4, for dihydrocapsaicin *m/z* 308.2 > 137.4, nordihydrocapsaicin *m/z* 294.2 > 137.4, homocapsaicin *m/z* 320.1 > 137.2 and homodihydrocapsaicin *m/z* 322.1 > 137.2. Homocapsaicin and homodihydrocapsaicin were expressed as capsaicin and dihydrocapsaicin equivalents, respectively. All five capsaicinoids are expressed in mg/100 g DW.

### Statistical analysis

Program R^15^ was used for statistical analysis. Where significant treatment effects were found using analysis of variance (ANOVA), the Tukey-test was performed. The significant level was α = 0.05. Pearson’s test for correlation (α < 0.05) among metabolites was performed using a correlation matrix to determine whether any metabolite affected another. Multivariate analyses PCA was performed to check how different cultivars are connected among each other in terms of different metabolites. Hierarchical clustering (dendrogram) using R was used to determine the grouping of different cultivars, using Ward’s method based on Euclidian distance. For 'Bhut Jolokia', only average values and standard errors are presented, due to lack of other *C. chinense* × *C. frutescens* cultivars, which would enable deeper statistical analysis. Total contents of all metabolites were calculated according to each fruit part share in the whole fruit.

## Results and discussion

### Fruits characteristic

**Figure 1:**
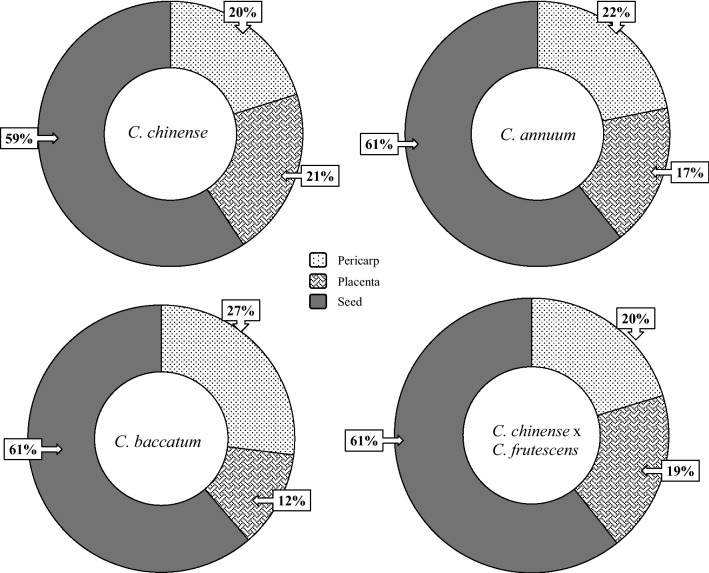
Average dry weight ratio among pericarp, placenta and seed in four different species of chilies.

The dry weight ratio of pericarp, placenta and seeds was evaluated. The results are presented in Fig. 1. Fresh weight data are shown in Fig. S1. In all four species, seeds had the highest dry weight among all three fruit parts and also similar % (from 59 to 61%). On average, seeds represented two thirds of the whole fruit dry weight. *C. chinense*, *C. annuum* and *C. chinense* × *C. frutescens* placenta and pericarp had similar dry weight ratios. In *C. baccatum* placenta had only 12% of the whole fruit dry weight. The highest amount of water was found in pericardp and placenta in all species. Cultivars with the most water content and, consequently, the biggest difference between fresh weight and dry weight, were 'Moruga Scorpion yellow' (10 times difference) in *C. chinense*, 'Jalapeño' (10 times difference) in *C. annuum* and 'Bishops Crown' (8 times difference) in *C. baccatum*. Chili fruits can contain from 85 to 95% water, as previously reported by Jarret and Berke^16^. The average weight of *C. chinense* fruits was 9.1 g*, C. annuum* 8.5 g, *C. baccatum* 5.6 g and *C. chinense* × *C. frutescens* 6.2 g. The highest fruit weight among all cultivars was determined in 'Jalapeno' (Table 1). Jarret and Berke^16^ report an average weight of 6.3 g for *C. chinense* fruits, which is in the range of our results. Nine cultivars had red fruits, 6 cultivars had yellow fruits and 1 each cultivar was colored brown, orange, white, pale yellow and yellowish green (Table 1).

**Table 1:** Fruit characteristics in different species and cultivars.

Species	Cultivar	Pericarp		Placenta		Seeds		Fruit mass (g)	Fruit color
		Fresh (%)	Dry (%)	Fresh (%)	Dry (%)	Fresh (%)	Dry (%)		
*C. chinense*	'Habanero Chocolate'	89	84	8	9	4	7	12.1 ± 0.6	Brown
	'Habanero Orange'	77	72	18	18	5	10	7.1 ± 0.2	Orange
	'Habanero Yellow'	86	80	11	11	4	9	13.3 ± 1.0	Yellow
	'Aribibi Gusano'	86	82	10	12	3	6	5.2 ± 0.2	White
	'Moruga Scorpion yellow'	89	80	8	8	3	12	12.9 ± 0.9	Yellow
	'Naga Morich'	78	71	13	10	9	18	3.7 ± 0.1	Red
	'7 Pot Primo Yellow'	86	85	9	5	5	10	9.0 ± 0.6	Yellow
	'Jay's Scorpion Peach'	84	81	11	8	5	11	8.8 ± 0.7	Pale yellow
	'Carolina Reaper'	89	72	7	13	4	14	8.3 ± 0.6	Red
	'Big Mustard Mama'	90	84	7	7	3	9	7.1 ± 0.4	Yellowish green
	'Borg 9 Pheno'	81	73	10	8	8	19	6.5 ± 0.2	Red
	'Yellow Cap Mushroom'	83	79	14	9	3	12	12.0 ± 1.1	Yellow
*C. annuum*	'Cayenne	87	76	7	3	6	21	4.8 ± 0.3	Red
	'Bolivian Rainbow'	83	67	9	5	8	27	4.9 ± 0.1	Red
	'Chilli AS- Rot'	71	60	14	8	15	32	11.8 ± 0.8	Red
	'Serrano'	75	70	9	9	16	21	5.4 ± 0.1	Red
	'Jalapeno'	83	73	7	8	10	19	21.3 ± 1.8	Green/Red
*C. baccatum*	'Aji Pineapple'	89	80	5	3	5	17	6.2 ± 0.2	Yellow
	'Bishops Crown'	85	81	8	2	7	17	6.8 ± 0.3	Red
	'Lemon Drop'	90	89	4	3	6	8	4.3 ± 0.1	Yellow
*C. chinense* × *C. frutescens*	'Bhut Jolokia'	89	84	7	6	4	10	6.2 ± 0.3	Red

### Sugars

**Figure 2:**
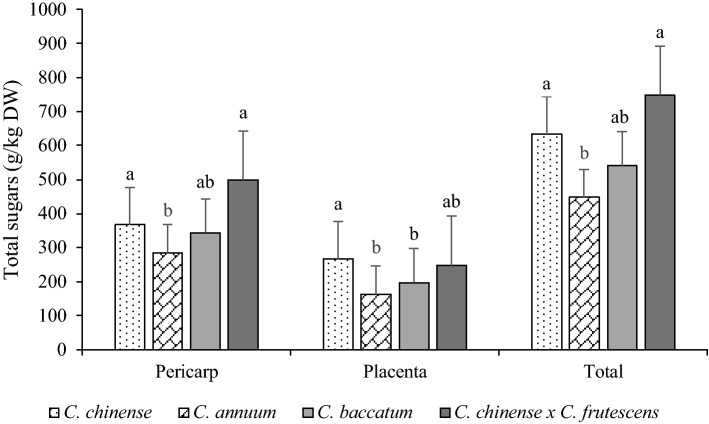
Total sugars (g/kg DW, mean ± SE) in three different species and two chili fruit parts. *a,b lower case letters denote statistical significant differences (α < 0.05) between total sugars in placenta, pericarp and whole fruit between species.

Sugars content in pericarp and placenta for each species is presented in Fig. 2. There are statistically significant differences between total sugars in the two fruit parts. *C.* *chin*e*nse* × *C. frutescens* and *C. chinense* had the largest average amount of total sugars in both parts (498.6 g/kg DW, 366.7 g/kg DW in pericarp and 248.5 g/kg DW and 266.5 g/kg DW in placenta) among all the studied species. The highest average content of total sugars was determined in *C. chinense* × *C. frutescens* fruits (747.1 g/kg DW) and the lowest in *C. annuum* (448 g/kg DW). Sucrose is the least abundant sugar in chilies, followed by glucose and fructose. In all four species, the pericarp had a higher content of total sugars than the placenta.

In all of the analyzed cultivars of chilies, (Table S1), fructose was the most abundant sugar, followed by glucose and sucrose for both pericarp and placenta. 'Bhut Jolokia' had the highest amount of fructose in the pericarp (338.2 g/kg DW). 'Jay's Scorpion Peach' had the highest content of fructose in the placenta (179.8 g/kg DW). 'Habanero Yellow' had the highest content of glucose in the pericarp (234.5 g/kg DW) and 'Jay's Scorpion Peach' in the placenta (146.4 g/kg DW). Glucose content was highest in 'Bolivian Rainbow' (43.2 g/kg DW) pericarp and 'Carolina Reaper' placenta (41.2 g/kg DW) among all studied cultivars. Variations in the content of free sugars are present among different chili cultivars, the reason being genotype, environment and fruit ripeness^17^. Jarret et al.^17^ and Perla et al.^3^ reported large variations in total and individual sugars in *C. chinense* and *C. baccatum* species. In 123 genotypes of *C. baccatum,* the species range in total sugars was from 41 to 700 mg/g DW and in *C. chinense* the variation was from 198 to 1543 mg/100 g fresh weight, which is in the same range as our results. Red chilies did not have higher sugar content than other colors. Sugar content increases with ripeness and it is not correlated with the color of the chili^18,19^, which corresponds with our results.

### Organic acids

Organic acid contents of different species are presented in Table 2. *C. annuum* had a statistically significant higher content of ascorbic acid in the pericarp (1441 mg/100 g DW) compared to the other two species. In the placenta, no statistical differences were found between *C. chinense* and *C. annuum*. *C. baccatum* had the lowest amount of ascorbic acid in placenta (290.6 mg/100 g DW). Citric acid concentrations were higher in pericarp of *C. chinense* than in placenta (684.6 mg higher) and other organic acids in *C. chinense* were lower in pericarp. In *C. annuum,* quinic acid was higher (81.3 mg/100 g DW) in pericarp and all other organic acids were lower than in placenta (Table 2). In *C. baccatum* quinic and succinic acid contents were higher in pericarp and all citric, malic and fumaric acids were higher in the placenta. *C. chinense* × *C. frutescens* had similar organic acid contents as *C. chinense.* There were no statistically significant differences between placenta and pericarp when comparing total organic acids. *C. annuum* had the highest content of total organic acids in pericarp and placenta, with 15% more than *C. chinense* × *C. frutescens,* 20% more than *C. chinense* and 72% more than *C. baccatum*.

**Table 2:** Organic acids contents (mg/100 g DW, mean ± SE) in different species and two different fruit parts.

Species/fruit part	Ascorbic acid		Citric acid		Malic acid		Quinic acid		Succinic acid		Fumaric acid		Oxalic acid		Total organic acids	
	Pericarp	Placenta	Pericarp	Placenta	Pericarp	Placenta	Pericarp	Placenta	Pericarp	Placenta	Pericarp	Placenta	Pericarp	Placenta	Pericarp	Placenta
*C. chinense*	1171.9 ± 58.5a*	487.7 ± 45.1b	4985.4 ± 143.2a	4300.8 ± 394.4b	2502.7 ± 159.6b	3508.9 ± 202.6a	1075.5 ± 92.0a	1152.0 ± 68.2a	23.7 ± 2.2b	27.8 ± 1.3a	7.2 ± 0.7b	13.0 ± 0.9a	13.9 ± 1.1b	37.5 ± 6.4a	9780.4 ± 397.8a	9527.6 ± 673.8a
*C. annuum*	1441.0 ± 32.1a	504.7 ± 23.6b	5613.2 ± 599.0a	5914.3 ± 455.8a	3309.9 ± 376.9a	3959.2 ± 397.5a	1291.9 ± 235.7a	1210.6 ± 274.6a	22.3 ± 2.7a	31.1 ± 3.3a	7.6 ± 1.0b	29.3 ± 12.9a	28.5 ± 1.5a	50.8 ± 5.2a	11,714.3 ± 1215.3a	11,700.0 ± 1198.1a
*C. baccatum*	546.9 ± 42.0a	290.6 ± 12.8b	4802.1 ± 266.2a	5866.7 ± 437.8a	584.9 ± 42.9b	1088.0 ± 123.3a	801.6 ± 83.4a	249.3 ± 29.4b	45.0 ± 6.1a	44.3 ± 4.6a	4.6 ± 0.4b	6.5 ± 1.1a	34.9 ± 1.9a	13.1 ± 2.9a	6785.1 ± 399.0a	7558.6 ± 672a
*C. chinens e *× *C. frutescens*	1109.4 ± 14.9a	625.7 ± 50.7b	4516.4 ± 118.7a	5105.9 ± 407.1a	2559.8 ± 19.9a	3515.8 ± 316.7a	1040.4 ± 58.1b	1435.7 ± 114.2a	16.2 ± 1.3b	24.4 ± 1.7a	7.0 ± 0.3b	16.0 ± 0.8a	/	28.6 ± 3.1	8139.7 ± 526.2a	10,126.5 ± 947.9a
*C. chinense*	A*	A	A	B	AB	A	AB	A	B	B	A	B	A	A	B	B
*C. annuum*	A	A	A	A	A	A	A	A	B	B	A	A	A	A	A	A
*C. baccatum*	B	A	A	A	B	B	B	B	A	A	A	B	A	A	C	B
*C. chinense *× *C. frutescens*	AB	A	A	AB	AB	AB	AB	A	B	B	A	AB	/	A	BC	AB

*^a,b^Lower case letters denote statistical significant differences (α < 0.05) among placenta and pericarp of individual organic acid and total organic acids in the same species.

*^A–C^Upper case letters in same column denote statistical significant differences (α < 0.05) in organic acid concentrations among four different species separated for pericarp and placenta.

The organic acid contents of individual cultivars are presented in Tables S2 and S3. Oxalic acid was not detected in all chilies. In all different species, ascorbic, citric, malic and quinic acid were the most abundant. 'Bolivian Rainbow' had the highest content of citric acid in pericarp (94.8 g/kg DW) and placenta (105.8 g/kg DW) among all cultivars. 'Serrano' had the highest content of malic acid in pericarp (77.2 g/kg DW) and in placenta (59.0 g/kg DW) and fumaric acid in both fruit parts (15.8 mg/100 g and 78.8 mg/100 g DW). 'Cayenne' had the highest amounts of quinic acid invg both fruit parts. Succinic acid was most abundant in the pericarp of 'Aji Pineapple' (57.3 mg/100 g DW) and in the placenta of 'Bishops Crown' (59.5 mg/100 g DW). Citric acid is the most abundant organic acid in chilies and other acids can vary in content. Each chili cultivar can have a different organic acid profile^17,20^. Jarret et al.^17^ reported that organic acids in chili varieties depending on the genotype and environmental conditions. Environmental stress and genotype variation can reduce or increase the contents of certain organic acids^21,22^. Organic acid contents are also affected by the maturity stage of the fruit. Green fruits have less organic acids than fully developed, red fruits^23^.

Ascorbic acid contents for individual cultivars are presented in Fig. S2. Significant variations were present in ascorbic acid content. Similar variations in ascorbic acid content were reported by Antonious et al.^20^, Campos et al.^24^ and Teodoro et al.^25^ In *C. chinense* species, the highest ascorbic acid values were present in 'Aribibi Gusano' (1640 mg/100 g DW in pericarp and 985.9 mg/100 g DW in placenta), 'Habanero Orange' (1663.1 mg/100 g DW in pericarp and 821.7 mg/100 g DW in placenta). Of *C. annuum* species, 'Cayenne' had the highest amount of ascorbic acid in the pericarp (2105.6 mg/100 g DW) and 'Bolivian Rainbow' had the highest content of ascorbic acid in placental tissue (1009 mg/100 g DW). For *C. baccatum* species, the highest content of ascorbic acid was analyzed in 'Bishops Crown' for both pericarp and placenta (721.5 mg/100 g DW and 406.8 mg/100 g DW). Kumar and Tata^18^ and Korkutata and Kavaz^26^ reported that there were large differences between *C. annuum* chili cultivars and also between *C. baccatum*, as reported by Perla et al.^3^ Each chili in our experiment had a higher content of ascorbic acid in the pericarp than in the placenta. Ascorbic acid synthesis and accumulation is prevalent in the pericarp, as previously reported by Castro-Concha et al.^27^ Variation in different chilies in ascorbic acid content was reported by Jarret et al.^17^, the reason being genotype variation. Our results can be compared to Jarret et al.^17^, since we excluded all environmental factors and only genotype could have had an effect on the contents of ascorbic and other organic acids. Color did not affect the organic acid composition of fruits^28^ and Matsufuji et al.^19^ reported similar results, in which ripeness had a greater influence on organic acid content in fruits. Luning et al.^28^ also reported that citric acid has a noticeable effect on the taste of the fruit, combined with other substances.

### Total phenolic content

Total phenolic content for each species and fruit part is presented in Table 3. On average, the *C. chinense* species had 2183 mg/100 g DW of total phenolic in pericarp, 3004.3 mg/100 g DW in placenta and 478.2 mg/100 g DW in seeds. *C. chinense* × *C. frutescens* had 2125.6 mg/100 g DW in pericarp, 3607.0 mg/100 g DW in placenta and 467.8 mg/100 g DW in seeds. *C. annuum* species had 1626.6 mg/100 g DW in pericarp, 1396.9 mg/100 g DW in placenta and 239 mg/100 g DW in seeds. *C. baccatum* had on average the lowest content of total phenols in pericarp. On average, placenta had the highest content of total phenolics in *C. chinense*, *C. chinense* × *C. frutescens* and *C. baccatum*, followed by pericarp and seeds. *C. annuum* had a higher content of total phenolics in pericarp, followed by placenta and seeds. The total phenolic content of whole fruit was determined, based on the ratio of each fruit part to the whole fruit. If consuming a whole fruit, the most phenolics you can consume are from *C. chinense* and *C. chinense* × *C. frutescens*. Castro-Concha et al.^29^, reported similar results in which placenta also had the highest amount of total phenolics compared to other fruit parts. *C. annuum* species have less total phenolics than *C. chinense*^30^, which corresponds with our results.

**Table 3:** Total phenolic content (mg GAE/100 g DW, mean ± SE) in different species and three fruit parts.

Species/Fruit part	Pericarp	Placenta	Seed	Total phenolic content
*C. chinense*	2183.0 ± 116.1b*	3004.3 ± 118.7a	478.2 ± 45.5c	1349.6 ± 100.3a
*C. annuum*	1626.6 ± 144.8a	1396.9 ± 85.2a	239.0 ± 17.7b	741.1 ± 48.9b
*C. baccatum*	973.5 ± 65.9b	1582.6 ± 84.3a	241.3 ± 15.6b	600.0 ± 50.8b
*C. chinense *× *C. frutescens*	2125.6 ± 110.7b	3607.0 ± 333.8a	467.8 ± 39.4c	1395.8 ± 121.7a
*C. chinense*	A*	A	A	A
*C. annuum*	BC	B	B	B
*C. baccatum*	C	B	B	B
*C. chinense *× *C. frutescens*	AB	A	AB	A

*^a–c^Lower case letters denote statistical significant differences (α < 0.05) among placenta, pericarp and seeds of total phenolics in same species.

*^A–C^Upper case letters in same column denote statistical significant differences (α < 0.05) in total phenolics among placenta, pericarp, seeds and whole fruit in four different species.

The total phenolic content for individual cultivars is presented in Table S4. In *C. chinense* species, 'Borg 9 Pheno' and 'Carolina Reaper' had the highest amount of total phenolics in pericarp. In placenta and seeds, the most significant amount of total phenols was found in 'Carolina Reaper' and 'Aribibi Gusano'. 'Cayenne' had the highest content of total phenols (2553.8 mg/100 g DW) in pericarp compared to all cultivars in the *C. annuum* species in this experiment. It also had the highest content of phenols in the placental tissue and seeds. 'Bishops Crown' of the *C. baccatum* species had a high content of total phenolics in pericarp, but the least total phenols in placental tissue and in seeds compared to the other two cultivars. *C. chinense* × *C. frutescens* 'Bhut Jolokia' had 2125.6 mg/100 g DW of total phenols in pericarp, 467.8 mg/100 g DW in seeds and 3607 mg/100 g DW in placental tissue, which is the second highest content in the placenta of all tested cultivars. Noticeable variation is present in total phenolic content among different cultivars of chilies. Similar results were reported by Kumar et al.^31^ and Antonious et al.^20^, in which large variations among different genotypes were present, which corresponds with our results. Total phenolic content is also dependent on the maturity stage of the fruit. In later stages of maturity, total phenolic content becomes lower in chilies^32^. Red fruits in our experiment had higher phenolics content than other colors. Similar results were also reported by Ghasemnezhad et al.^32^ in which the red cultivar also had the highest total phenolic content. The difference among total phenolics is related to genotype and also the ripeness stage of the fruit itself. Apparently, red cultivars are liable to a higher total phenolic content.

### Capsaicinoids

**Figure 3:**
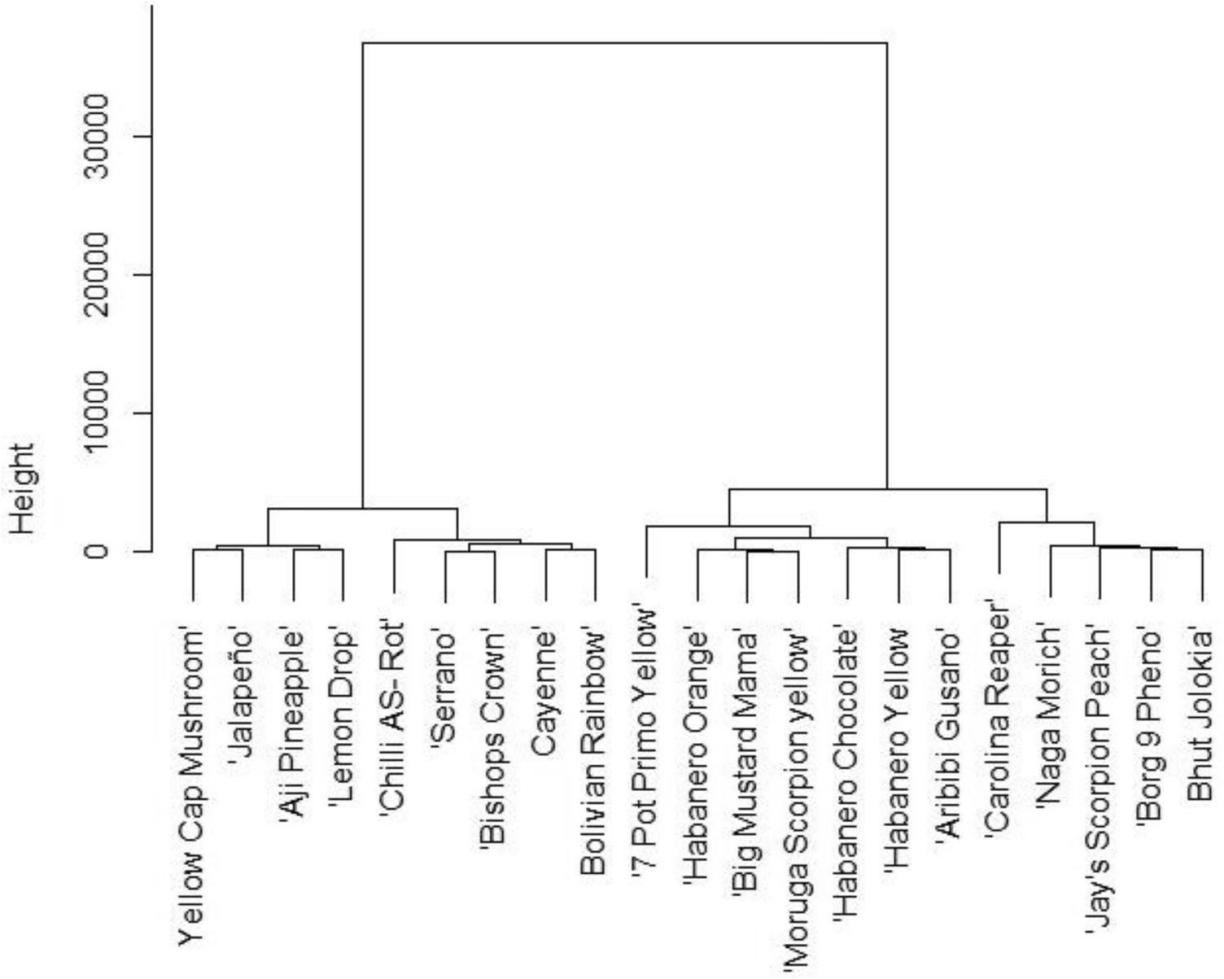
Clustering of chili cultivars based on total capsaicinoid contents.

The chili cultivars from our experiment were grouped by total capsaicinoid content (Fig. 3). Two groups and four subgroups formed. The first main group consisted of all *C. annuum* and *C. baccatum* cultivars and one *C. chinense* cultivar, which had the lowest content of total capsaicinoids of all the *C. chinense* species. The second main group consisted of *C. chinense* and *C. chinense* × *C. frutescens*. All second group cultivars can be ranked from high heat to extremely high heat levels. The dendrogram also shows how one group of the most pungent chilies ('Carolina Reaper', 'Borg 9 Pheno', 'Bhut Jolokia',… mainly contains red cultivars. Previous reports by Krajayklang et al.^33^ could not determine that color was related to chili pungency.

**Figure 4:**
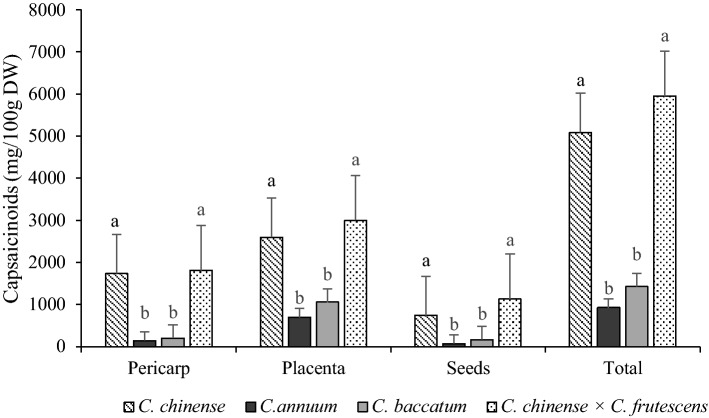
Total capsaicinoids (mg/100g DW, mean ± SE) in three different fruit parts and total capsaicinoids variations among four different species of chilies. *a,b lower case letters denote statistical significant differences (α < 0.05) among total capsaicinoids in placenta, pericarp, seeds and whole fruit among four different species.

The total capsaicinoid content of each individual fruit part and total capsaicinoid content for entire fruit are presented in Fig. 4. Statistically significant differences were present in all three fruit parts in terms of total capsaicinoid content. *C. chinense* and *C. chinense* × *C. frutescens* had the highest content of capsaicinoids in pericarp (1738 mg/100 g DW and 1809.2 mg/100 g DW), followed by placenta (2600 mg/100 g DW and 3004.3 mg/100 g DW) and seeds (748.7 mg/100 g DW and 1135.4 mg/100 g DW). The lowest content was determined in *C. annuum* fruit parts. *C. chinense* and *C. chinense* × *C. frutescens* contained from 3.5 to 5 times more capsaicinoids than the other two species. Placenta had the highest content of capsaicinoids followed by pericarp and seeds in all species. Wahyuni et al.^34^ reported similar results, in which the highest content of capsaicinoids was accumulated in placenta followed by pericarp and seeds. High contents of capsaicinoids accumulate in placenta, mostly capsaicin. The *C. chinense* species has the largest content of capsaicinoids in the entire *Capsicum* genus^5^.

In *C. chinense* pericarp, 'Carolina Reaper' had the highest content of capsaicin (2443.8 mg/100 g DW), dihydrocapsaicin (644.4 mg/100 g DW), nordihydrocapsaicin (126.1 mg/100 g DW) and homodihydrocapsaicin (62.5 mg/100 g DW) and 'Naga Morich' the highest content of homocapsaicin (78.1 mg/100 g DW) (Table S5). In placenta, 'Naga Morich' had the highest content of homocapsaicin (156.8 mg/100 g DW) and 'Carolina Reaper' had the highest content of homodihydrocapsaicin (92.6 mg/100 g DW). Capsaicin in placenta was highest in 'Habanero Chocolate' (2522 mg/100 g DW). Dihydrocapsaicin (580.3 mg/100 g DW) and nordihydrocapsaicin (178 mg/100 g DW) content was highest with 'Habanero Orange' (Table S6). In seeds, the highest content of individual capsaicinoids was present in 'Carolina Reaper' and 'Naga Morich' (Table S7).

'Cayenne' from the *C. annuum* species, had the highest content of capsaicin, nordihydrocapsaicin, homocapsaicin and homodihydrocapsaicin. 'Serrano' had the highest content of dihydrocapsaicin in pericarp (Table S5). 'Jalapeño' had the highest content of all five studied capsaicinoids in placenta and the lowest content was in the cultivar 'Chili AS- Rot' (Table S6). 'Jalapeño' also had the highest content of all capsaicinoids except for homodihydrocapsaicin, which was most abundant in 'Cayenne' seeds (5.6 mg/100 g DW) (Table S7).

In *C. baccatum*, 'Aji Pineapple' had the highest content of capsaicin (246.7 mg/100 g DW) and dihydrocapsaicin (35.5 mg/100 g DW) and 'Lemon Drop' of nordihydrocapsaicin, homocapsaicin and homodihydrocapsaicin (Table S5). In placenta, 'Aji Pineapple' had the highest content of dihydrocapsaicin, nordihydrocapsaicin and homocapsaicin and 'Lemon Drop' of capsaicin and homodihydrocapsaicin (Table S6). In seeds, 'Aji Pineapple' had the highest content of all five capsaicinoids (Table S7).

*C. chinense* × *C. frutescens* 'Bhut Jolokia' had the highest content of capsaicinoids in placenta (Table S6). It had one of the highest contents of capsaicin and homodihydrocapsaicin in placenta. In seeds, it had a similarly high content of capsaicin (1011.4 mg/100 g DW) as the hottest cultivar 'Carolina Reaper' (1090 mg/100 g DW) (Table S7). Gnayfeed et al.^35^ and Wahyuni et al.^34^ reported a noticeable variation among different cultivars. Reasons for variations are environmental factors, genotype factors, fruit maturity, irrigation water regimes and enzymatic activity^10,36^.

The capsaicinoid composition of the pericarp was on average 82% capsaicin, 13.1% dihydrocapsaicin, 2.2% nordihydrocapsaicin, 1.7% homocapsaicin and 1% homodihydrocapsaicin. The composition of the placenta was 77.5% capsaicin, 11.9% dihydrocapsaicin, 5% nordihydrocapsaicin, 3.2% homocapsaicin and 2.5% homodihydrocapsaicin. The seeds contained 83.6% capsaicin, 12.2% dihydrocapsaicin, 2% nordihydrocapsaicin, 1.4% homocapsaicin and 0.8% homodihydrocapsaicin. Capsaicin and dihydrocapsaicin account for 85% to 95% of all capsaicinoids in chilies, as already reported by Jeeatida et al.^37^ and this is consistent with our results. The placenta is the main synthesis point for capsaicinoids. Capsaicinoids are concentrated in oil cells located in the placenta that supports the seed^2^. Other parts of the fruit, such as the pericarp and seeds, can also synthesize capsaicinoids, although in much smaller quantities, since the enzymes for their synthesis are also present in other parts of the fruit^38^. Chili seeds have the lowest content of capsaicinoids in all cultivars. The seeds are important for the continuation of the plant's life cycle. Capsaicin and other capsaicinoids have an inhibitory role. Seeds of chilies treated with capsaicin germinated more slowly^39^. Capsaicin can delay or stop germination completely. One aim of the plants is to protect seeds with the pericarp and placenta, which have a high content of capsaicinoids, but the seeds have a low content so that they can germinate more quickly^7^. Hot peppers are mostly consumed fresh, dried or in powder form (50–70%) mainly in the agro-food industry^40^. Pepper extract (E 160c) is classified as a food additive (natural coloring) in the EU^41^. Thirty percent of peppers are wastes, such as peels, seeds, stems, and unused pericarp or placenta, which can be used as a source of secondary metabolites such as phenols and capsaicnoids in the pharmaceutical industry, which uses them for the main source of capsaicnoids for neurological and musculoskeletal pain, inflammatory and oxidative disease states, etc^42,43^. Peppers suitable for pharmaceutical industry in our case would be 'Carolina Reaper', 'Borg 9 Pheno', 'Bhut Jolokia', etc. with high capsaicnoid content. In the cosmetic industry, peppers are used for their natural coloration, texture or flavor of the final product, which makes them safer for the consumer^40^. Knowing how the metabolites are distributed among the different parts of the fruit in the different cultivars and species, we could reduce waste and optimize industrial efficiency, since peppers are used in several industries.

### Correlation between metabolites and cultivars

**Figure 5:**
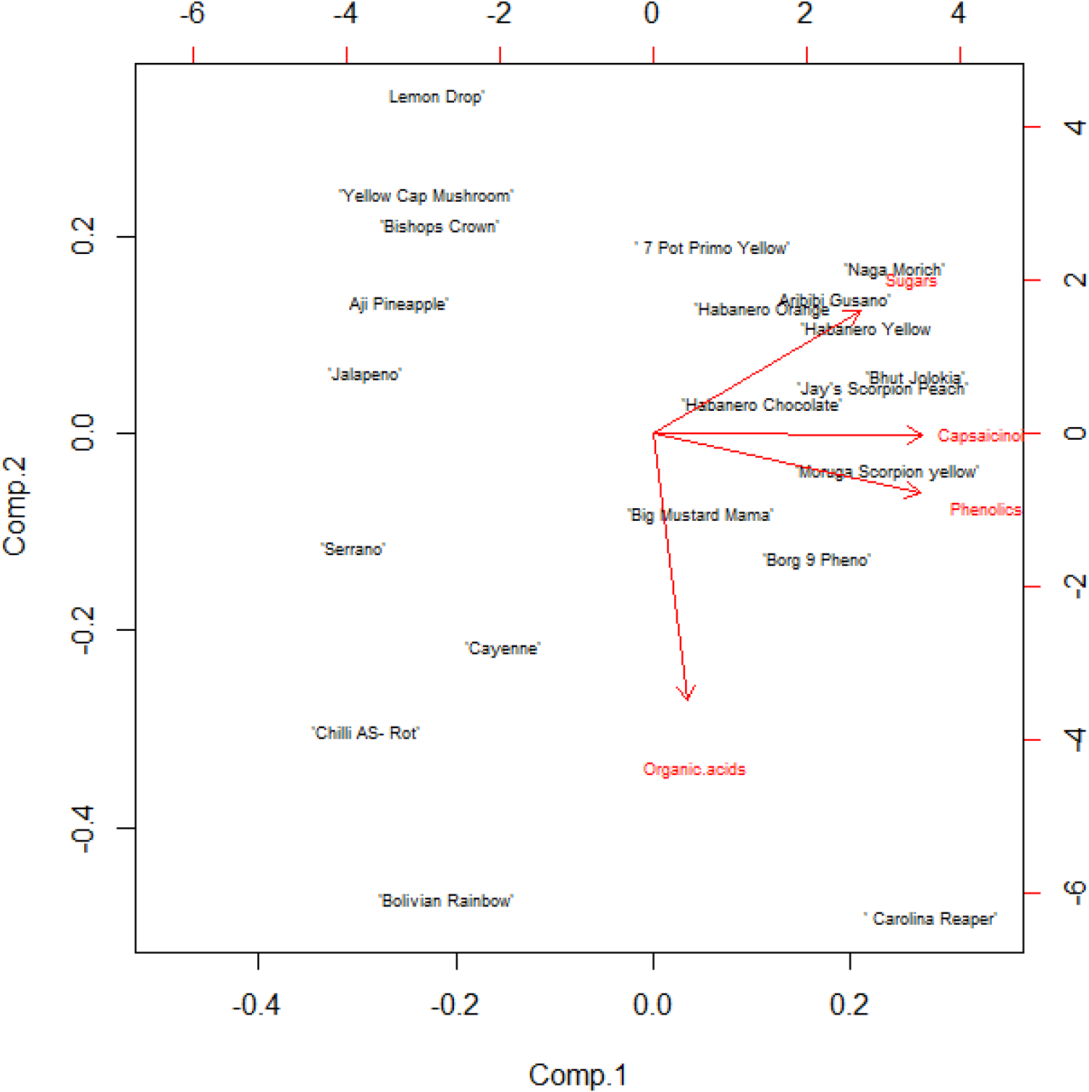
PCA analysis of all cultivars.

PCA analysis was performed to gain better insight into the intra and inter species variation in terms of metabolites (Fig. 5). The results of PCA analysis showed a large cluster of cultivars consisting mainly of the *C. chinense* species, which had high capsaicinoid, phenolic and sugar contents. On the other hand, most of the *C. baccatum* and *C. annuum* cultivars had lower metabolite contents. Among all the cultivars, the cultivar 'Carolina Reaper' was the most interesting with high content of capsaicinoids, phenols, sugars and organic acids. The cultivar 'Bolivian Rainbow' also had a high content of organic acid compared to the other cultivars.

A correlation test was made between different metabolites to determine whether they are in any way connected. Total sugars amount was statistically significantly correlated to total phenolic content (p = 0.0162) and total capsaicinoid content (p = 0.0007). Total phenolic content and total capsaicinoid content were strongly correlated (*p* < 0.0001). Materska and Perucka^44^ reported that a strong correlation is present among total phenolics and capsaicinoids. The correlation between phenolics and capsaicinoids could be the result of their biosynthetic pathway. Both groups of metabolites use phenylalanine (result of the phenylpropanoid pathway) as the substrate for biosynthesis^30^, which in chilies can result in an even distribution of phenylalanine between phenolics synthesis and capsaicinoids synthesis, resulting in a similar metabolite level in the same cultivar. Phenylalanine synthesis increases in later stages of fruit development and ripening, which results in higher secondary metabolite levels^8^.

## Conclusion

For maximum capsaicinoid consumption and industrial use of capsaicinoids, *C. chinense* or *C. chinense* × *C. frutescens* cultivars should be used. The largest impact on human health is provided by consuming placenta and pericarp who have the highest contents of several metabolites including capsaicinoids. Seeds have low nutritional value compared to other parts of the fruit. Choosing the right cultivar is crucial, as confirmed by our experiment. Our study had equal environmental factors, which increases the quality of our results. The UHPLC–PDA–QMS method is also very accurate and reliable for the determination of metabolites in different parts of the fruit. Our study gives a great insight into the metabolite composition of chili cultivars and species, for each fruit part separately, as the prices of chili may vary depending on their capsaicinoid content^16^ especially in pharmaceutical industry. The study provides excellent insight into the capsaicinoids and other metabolite profiles of various cultivars, twelve of which have not been reported previously. Cultivars like 'Carolina Reaper', 'Cayenne', 'Jalapeño' etc. could be considered for intensive production in Southern and Central Europe, for food, pharmaceuticals or used with other industries due to their high overall metabolite contents.

### Statements on plant material

The plant material in this manuscript complies with relevant institutional, national and international guidelines and laws and can be obtained from various seed companies worldwide.

## Supplementary Information

Supplementary Information
