# Supplementary material for: Influence of intra and inter species variation in chilies (Capsicum spp.) on metabolite composition of three fruit segments
Source: Sci Rep. 2021 Mar 2;11:4932. doi: 10.1038/s41598-021-84458-5 (PMC7925605; doi:10.1038/s41598-021-84458-5)
Supplement: Supplementary file 1 — Supplementary Information [file 41598_2021_84458_MOESM1_ESM.pdf]

Influence of intra and inter species variation in chilies (*Capsicum* spp.) on metabolite composition of three fruit segments.

Tilen ZAMLJEN [a*], Jerneja JAKOPIČ [a], Metka HUDINA [a], Robert VEBERIČ [a], Ana SLATNAR [a]

Authors Affiliation:

[a] University of Ljubljana, Biotechnical Faculty, Department of Agronomy, Jamnikarjeva 101, SI-1000 Ljubljana, Slovenia

[*]Corresponding author: tilen.zamljen@bf.uni-lj.si

jerneja.jakopic@bf.uni-lj.si

metka.hudina@bf.uni-lj.si

robert.veberic@bf.uni-lj.si

ana.slatnar@bf.uni-lj.si

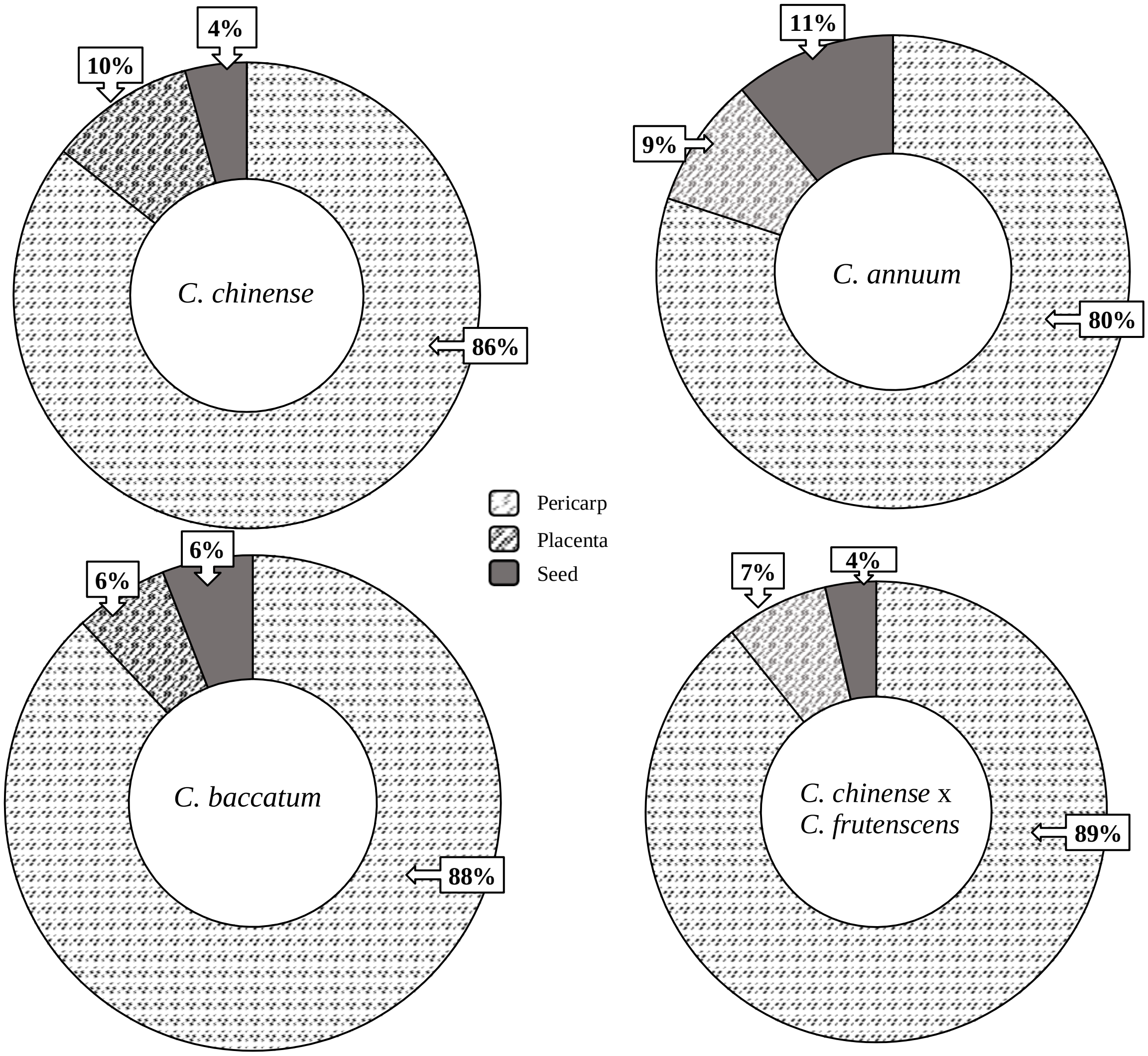

Figure S1: Average fresh weight ratio among pericarp, placenta and seed in four different species of chilies.

Table S1: Individual sugars (g/kg DW, mean ± SE) in different chili cultivars and two chilie fruit parts.

Species	Cultivar/fruit part	Fructose				Glucose				Sucrose			
		Pericarp		Placenta		Pericarp		Placenta		Pericarp		Placenta	
C. chinense	'Habanero Chocolate'	179.8 ± 21.4	abcd*	142.0 ± 5.3	c	174.4 ± 21.1	abc	93.3 ± 25.6	b	22.8 ± 2.8	ab	34.5 ± 2.6	a
	'Habanero Orange'	225.4 ± 18.9	abc	138.8 ± 28.2	c	175.0 ± 15.7	abc	40.3 ± 15.9	c	7.1 ± 1.3	b	28.6 ± 2.2	ab
	'Habanero Yellow'	247.9 ± 27.2	a	174.2 ± 7.9	b	234.5 ± 29.6	a	97.6 ± 28	ab	10.3 ± 0.3	b	32.1 ± 2.4	a
	'Aribibi Gusano'	239.7 ± 9.2	ab	139.2 ± 32	c	200.6 ± 8	ab	65.6 ± 5.2	c	34.0 ± 5.8	a	31.8 ± 0.6	a
	'Moruga Scorpion yellow'	215.7 ± 3.2	abcd	170.7 ± 12	b	162.3 ± 13	abcd	124.9 ± 3	ab	30.5 ± 2.4	a	39.5 ± 1.9	a
	'Naga Morich'	188.8 ± 5.6	abcd	184.8 ± 3.2	a	157.5 ± 14.6	abcd	143.7 ± 8.6	a	20.8 ± 7.3	ab	29.3 ± 3.9	ab
	'7 Pot Primo Yellow'	204.0 ± 13.3	abcd	157.0 ± 12.5	bc	154.5 ± 18.2	abcd	86.7 ± 8.9	b	30.9 ± 2.4	a	28.3 ± 4.1	ab
	'Jay's Scorpion Peach'	170.7 ± 17.3	bcd	179.8 ± 27.6	b	174.3 ± 10.6	abc	146.4 ± 5	a	34.9 ± 2.5	a	39.6 ± 7.3	a
	'Carolina Reaper'	165.3 ± 4.6	cd	96.8 ± 10.6	d	91.1 ± 16	cd	43.4 ± 16.5	c	37.6 ± 4.3	a	41.2 ± 2.4	a
	'Big Mustard Mama'	164.3 ± 13.7	cd	85.0 ± 3.5	d	124.3 ± 50.2	abcd	87.3 ± 29.3	b	36.2 ± 3.8	a	34.2 ± 3.3	a
	'Borg 9 Pheno'	164.3 ± 8.2	d	85.1 ± 4.2	d	66.2 ± 21	cd	68.9 ± 25.8	c	34.3 ± 0.8	a	24.1 ± 4.6	b
	'Yellow Cap Mushroom'	145.5 ± 5.5	d	140.6 ± 3	c	78.8 ± 34.6	bcd	97.5 ± 16.5	ab	34.1 ± 2.8	ab	32.4 ± 2.4	a
C. annuum	'Cayenne	136.9 ± 1.2	ab	99.5 ± 3	ab	117.9 ± 8	a	67.7 ± 6.9	ab	36.1 ± 2.5	a	33.1 ± 0.3	a
	'Bolivian Rainbow'	137.0 ± 6.7	ab	72.6 ± 1.7	ab	106.4 ± 2.1	ab	61.8 ± 8.7	ab	43.2 ± 2	a	31.7 ± 4.3	a
	'Chilli AS- Rot'	174.1 ± 8.1	a	57.8 ± 0.5	ab	127.2 ± 8.5	a	35.9 ± 3.6	b	38.6 ± 1.1	a	35.8 ± 2.9	a
	'Serrano'	93.6 ± 9.8	b	101.6 ± 9	a	86.1 ± 6.5	b	83.5 ± 4.4	a	22.5 ± 1.3	b	25.7 ± 5.3	a
	'Jalapeno'	117.4 ± 1.4	b	45.0 ± 7.4	b	151.9 ± 7.4	a	33.2 ± 0.4	b	34.7 ± 2.2	a	31.1 ± 4.7	a
C. baccatum	'Aji Pineapple'	158.7 ± 4	b	72.3 ± 2.9	b	107.0 ± 3.4	b	59.6 ± 2.3	ab	31.4 ± 4.2	a	26.3 ± 4.8	ab
	'Bishops Crown'	198.6 ± 6.6	a	110.9 ± 8.5	a	152.7 ± 8.9	a	74.1 ± 3.4	a	28.2 ± 1.4	a	40.6 ± 3.4	a
	'Lemon Drop'	170.0 ± 5.3	b	101.0 ± 7	a	154.3 ± 12.4	a	79.1 ± 3.3	a	29.7 ± 3.2	a	15.0 ± 4.8	b
C. chinense x *C. frutescens*	'Bhut Jolokia'	338.2 ± 14.2	/	127.0 ± 5.2	/	124.3 ± 11.2	/	87.3 ± 5.8	/	36.2 ± 3.1	/	34.2 ± 2.9	/

*a - d lower case letters denote statistical significant differences (α < 0.05) among cultivars of the same species in the same column for placenta and pericarp.

Table S2: Organic acids (g/kg DW, mean ± SE) in different cultivars and two different fruit parts.

Species	Cultivar/ fruit part	Citric acid				Malic acid				Quinic acid			
		pericarp		placenta		pericarp		placenta		pericarp		placenta	
C. chinense	'Habanero Chocolate'	53.6 ± 1.9	bc*	41.2 ± 4.0	bcd	28.6 ± 1.5	abcd	38.0 ± 3.1	abcd	7.9 ± 0.6	b	10.1 ± 0.3	bcd
	'Habanero Orange'	27.9 ± 1.7	f	27.0 ± 1.3	d	22.8 ± 1.3	cde	43.0 ± 1.7	ab	16.2 ± 1.7	a	15.1 ± 1.1	a
	'Habanero Yellow'	52.0 ± 3.1	c	34.1 ± 2.3	d	34.1 ± 2.4	ab	38.0 ± 2.6	ab	13.5 ± 0.8	ab	9.6 ± 0.2	cd
	'Aribibi Gusano'	57.9 ± 1.2	bc	42.2 ± 3.3	bcd	14.5 ± 0.4	e	24.0 ± 0.9	d	10.9 ± 0.9	ab	11.1 ± 1.0	abc
	'Moruga Scorpion yellow'	55.2 ± 2.7	bc	51.3 ± 0.8	bc	31.2 ± 1.2	abc	42.6 ± 0.9	ab	10.1 ± 1.0	ab	12.6 ± 0.6	abc
	'Naga Morich'	38.0 ± 1.6	de	34.0 ± 1.5	d	27.2 ± 1.3	abcd	35.4 ± 1.7	abcd	9.0 ± 0.1	b	12.9 ± 0.5	abc
	'7 Pot Primo Yellow'	40.7 ± 0.7	d	33.1 ± 2.2	d	18.9 ± 0.9	de	32.3 ± 2.0	abcd	11.7 ± 0.7	ab	10.9 ± 1.2	abc
	'Jay's Scorpion Peach'	61.7 ± 1.7	b	57.4 ± 5.8	b	19.8 ± 2.3	de	28.8 ± 0.6	bcd	7.8 ± 0.3	b	11.1 ± 0.9	abc
	'Carolina Reaper'	78.8 ± 0.8	a	77.8 ± 3.4	a	27.5 ± 2.8	abcd	44.5 ± 0.9	a	10.3 ± 0.5	ab	13.0 ± 1.1	abc
	'Big Mustard Mama'	51.4 ± 1.4	c	39.7 ± 1.8	cd	25.6 ± 1.5	bcd	31.5 ± 1.3	abcd	8.3 ± 0.8	b	13.9 ± 1.0	ab
	'Borg 9 Pheno'	50.3 ± 1.1	c	40.9 ± 2.2	bcd	36.2 ± 2.3	a	38.7 ± 0.6	abc	10.3 ± 0.0	ab	12.0 ± 1.4	abc
	'Yellow Cap Mushroom'	30.8 ± 1.5	ef	37.3 ± 3.6	cd	13.8 ± 0.6	e	24.0 ± 1.1	cd	13.0 ± 1.2	ab	5.9 ± 0.7	d
C. annuum	'Cayenne'	51.5 ± 2.3	b	58.9 ± 3.7	b	22.7 ± 2.4	b	38.8 ± 2.3	b	20.6 ± 1.7	a	22.2 ± 2.0	a
	'Bolivian Rainbow'	94.8 ± 8.9	a	105.8 ± 9.3	a	22.7 ± 2.0	b	22.9 ± 1.4	b	14.3 ± 0.6	a	8.1 ± 0.5	ab
	'Chilli AS- Rot'	63.4 ± 6.0	ab	76.4 ± 3.4	b	25.8 ± 1.9	b	56.2 ± 2.3	a	11.8 ± 1.4	a	6.8 ± 0.1	ab
	'Serrano'	14.7 ± 1.2	c	14.8 ± 1.0	c	77.2 ± 5.8	a	59.0 ± 4.7	a	12.7 ± 1.2	a	10.3 ± 0.9	ab
	'Jalapeno'	56.3 ± 4.3	bc	39.8 ± 798	b	17.1 ± 1.6	b	21.1 ± 2.7	b	5.2 ± 0.2	a	13.1 ± 1.2	b
C. baccatum	'Aji Pineapple'	61.4 ± 1.9	a	58.9 ± 1.1	a	6.5 ± 0.7	a	9.7 ± 0.9	a	4.9 ± 0.1	b	1.2 ± 0.0	a
	'Bishops Crown'	40.2 ± 2.5	b	71.3 ± 1.1	a	7.0 ± 0.5	a	13.3 ± 1.4	a	11.3 ± 0.2	a	4.4 ± 1.0	a
	'Lemon Drop'	42.4 ± 3.5	b	45.8 ± 4.5	a	4.0 ± 0.3	b	9.6 ± 0.2	a	7.7 ± 0.1	ab	4.2 ± 0.9	a
C. chinense x *C. frutescens*	'Bhut Jolokia'	45.2 ± 1.2	/	51.0 ± 4.0	/	25.6 ± 1.9	/	35.2 ± 3.1	/	10.4 ± 0.3	/	14.2 ± 1.1	/

*a - f lower case letters denote statistical significant differences ($\alpha < 0.05$) among cultivars of same species for pericarp and placenta.

Table S3: Organic acids (mg/100g DW, mean ± SE) in different cultivars and two different fruit parts.

		Succinic acid				Fumaric acid				Oxalic acid			
Species	Cultivar/ fruit part	Pericarp		Placenta		Pericarp		Placenta		Pericarp		Placenta	
C. chinense	'Habanero Chocolate'	21.5 ± 0.1	b	30.2 ± 5.4	ab	8.0 ± 2.3	ab	16.5 ± 1.1	ab	/		44.9 ± 2.8	a
	'Habanero Orange'	21.7 ± 1.8	b	32.8 ± 2.0	a	8.3 ± 0.8	ab	20.4 ± 0.4	a	23.6 ± 5.7	a	19.1 ± 2.5	a
	'Habanero Yellow'	47.4 ±2.9	a	29.3 ± 6.5	b	8.2 ± 0.5	ab	10.4 ± 2.7	b	21.4 ± 10.3	a	13.7 ± 5.4	a
	'Aribibi Gusano'	14.8 ± 2.9	b	22.3 ± 1.5	b	5.6 ± 1.7	ab	11.7 ± 1.2	b	32.5 ± 16.8	a	50.0 ± 2.5	a
	'Moruga Scorpion yellow'	18.8 ± 0.7	b	22.5 ± 0.4	ab	7.9 ± 0.4	ab	15.2 ± 1.3	ab	/	/	30.5 ± 1.9	a
	'Naga Morich'	15.9 ± 0.8	b	31.0 ± 0.9	b	8.2 ± 0.6	ab	10.6 ± 1.1	a	2.6 ± 0.1	a	6.0 ± 0.2	a
	'7 Pot Primo Yellow'	20.3 ± 2.6	b	21.7 ± 0.9	ab	4.8 ± 0.7	ab	14.5 ± 1.7	ab	/	/	45.9 ± 1.3	a
	'Jay's Scorpion Peach'	29.5 ± 3.4	ab	34.2 ± 3.9	b	7.1 ± 0.3	ab	11.3 ± 0.5	b	4.0 ± 0.2	a	/	/
	'Carolina Reaper'	19.3 ± 0.2	b	29.4 ± 1.9	b	9.1 ± 0.3	a	11.4 ± 1	b	8.6 ± 0.9	a	88.5 ± 38.4	a
	'Big Mustard Mama'	20.8 ± 0.6	b	29.9 ± 0.2	b	6.5 ± 0.2	ab	13.3 ± 0.7	b	/	/	31.9 ± 0.7	a
	'Borg 9 Pheno'	25.9 ± 0.9	b	27.7 ± 1.3	b	9.1 ± 0.8	a	11.1 ± 1.6	b	4.9 ± 2.9	a	39.7 ± 1.7	b
	'Yellow Cap Mushroom'	28.9 ± 1.4	ab	22.6 ± 0.7	b	3.6 ± 0.4	b	9.9 ± 0.3	b	/	/	41.8 ± 7.4	a
C. annuum	'Cayenne	16.0 ± 3.6	b	20.3 ± 2.6	b	5.1 ± 0.4	b	24.4 ± 4.4	b	/	/	4.0 ± 1.4	a
	'Bolivian Rainbow'	40.8 ± 1.8	a	39.0 ± 1.3	a	6.5 ± 0.5	b	6.4 ± 0.4	b	45.1 ± 13.6	a	42.1 ± 2.3	a
	'Chilli AS- Rot'	19.0 ± 3.5	b	32.1 ± 5.7	b	5.3 ± 0.5	b	24.0 ± 7.0	b	/	/	49.1 ± 3.5	a
	'Serrano'	16.1 ± 1.9	b	22.2 ± 2.4	b	15.8 ± 3.4	a	78.8 ± 4.6	a	37.9 ± 10.6	a	97.9 ± 6.7	a
	'Jalapeno'	19.6 ± 3.5	b	41.9 ± 3.2	b	5.3 ± 0.2	b	12.8 ± 1.7	b	2.3 ± 0.4	a	60.8 ± 6.7	a
C. baccatum	'Aji Pineapple'	57.3 ± 5.9	a	36.5 ± 1.9	a	4.5 ± 0.3	a	4.9 ± 1.0	a	35.0 ± 7.8	a	9.6 ± 0.8	a
	'Bishops Crown'	53.6 ± 4.6	ab	59.5 ± 10.6	a	4.8 ± 0.3	a	5.9 ± 1.2	a	/	/	16.6 ± 0.8	a
	'Lemon Drop'	24.1 ± 0.8	b	37.0 ± 7.8	a	4.4 ± 0.7	a	8.6 ± 0.5	a	/	/	/	/
C. chinense x *C. frutescens*	'Bhut Jolokia'	16.2 ± 1.3	/	24.4 ± 1.7	/	7.0 ± 0.3	/	16.0 ± 0.8	/	/	/	28.6 ± 3.1	/

*a, b lower case letters denote statistical significant differences ($\alpha < 0.05$) among cultivars of same species for pericarp and placenta.

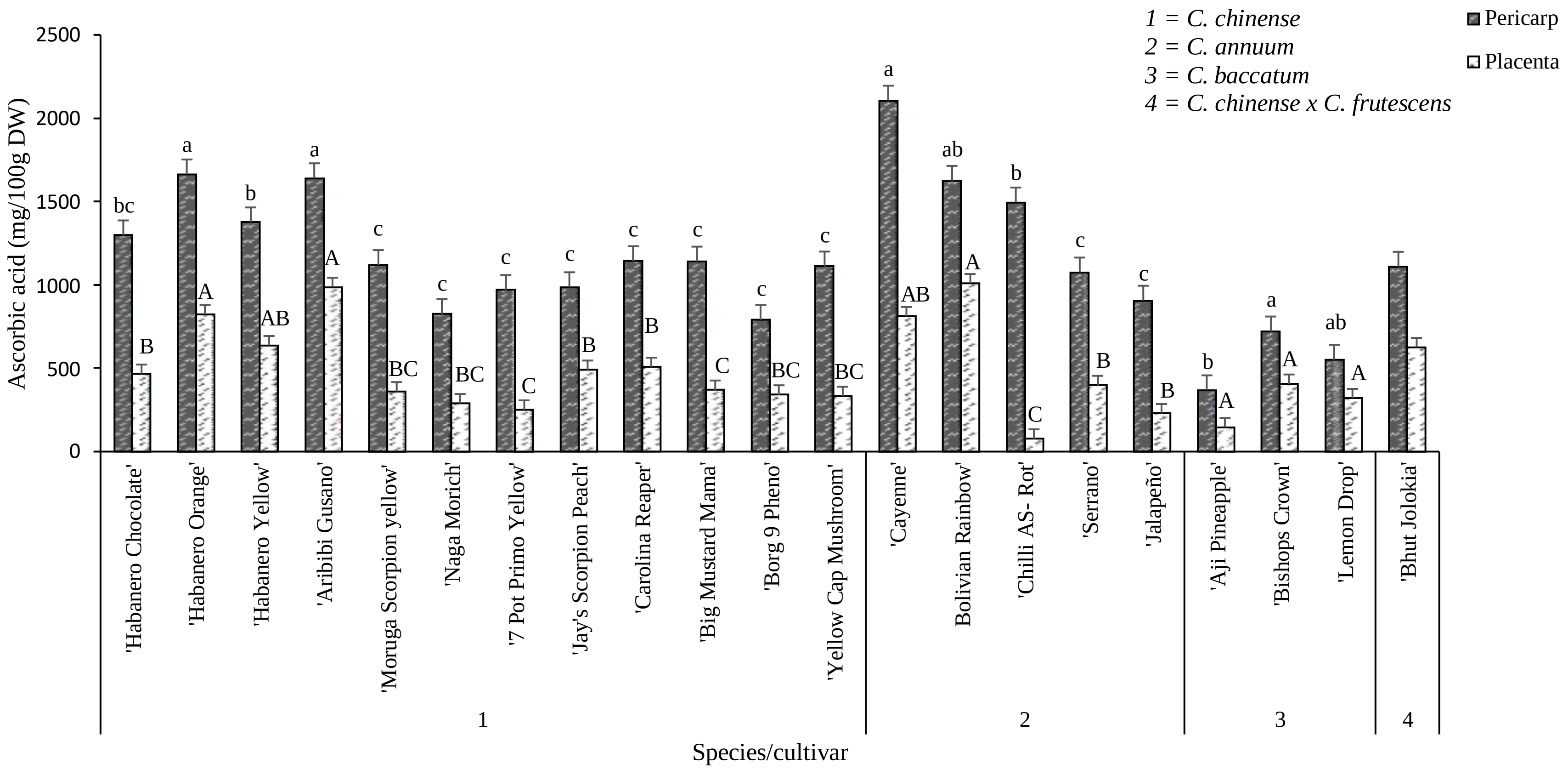

Figure S2: Ascorbic acid content in placenta and pericarp in different chili cultivars and species. *a - c denote statistical differences among varieties pericarp and *A – C denote statistical differences between culitvars placenta in the same species.

Table S4: Total phenolic content (mg GAE/100 g DW, mean ± SE) in different cultivars and in three fruit parts.

Species	Cultivar/fruit part	Pericarp		Placenta		Seed	
C. chinense	'Habanero Chocolate'	1842.7 ± 137.0	cde*	2760.1 ± 211.0	ab	435.3 ± 61.0	ab
	'Habanero Orange'	2158.9 ± 123.2	cde	2758.4 ± 121.8	ab	487.7 ± 53.5	a
	'Habanero Yellow	1854.1 ± 106.8	cde	3244.6 ± 288.4	a	529.0 ± 50.8	a
	'Aribibi Gusano'	1934 ± 121.7	cde	3433.9 ± 110.5	a	582.1 ± 46.9	a
	'Moruga Scorpion yellow'	2446.4 ± 262.0	bc	3211.2 ± 87.9	a	386.7 ± 25.0	ab
	'Naga Morich'	2221.7 ± 230.7	cd	3276.5 ± 274.4	a	490.8 ± 59.1	a
	'7 Pot Primo Yellow'	1304.8 ± 82.7	e	3101.9 ± 416.7	a	543.9 ± 44.0	a
	'Jay's Scorpion Peach'	2180.3 ± 97.9	cde	3166.8 ± 176.6	a	444.7 ± 38.6	ab
	'Carolina Reaper'	3322.8 ± 370.8	ab	3874.7 ± 259.0	a	569.2 ± 45.1	a
	'Big Mustard Mama'	2049.1 ± 109.2	cde	3211.2 ± 349.6	a	507.0 ± 30.1	a
	'Borg 9 Pheno'	3528.7 ± 89.9	a	2505.7 ± 85.8	ab	505.0 ± 59.8	a
	'Yellow Cap Mushroom'	1352.5 ± 62.6	de	1506.7 ± 164.4	b	256.8 ± 2.3	b
C. annuum	'Cayenne'	2553.8 ± 40.7	a	1685.4 ± 125	a	293.1 ± 52.2	a
	'Bolivian Rainbow'	1765.4 ± 147.7	b	1588.3 ± 17.4	a	217.3 ± 22.0	a
	'Chilli AS- Rot'	1524.8 ± 39.5	bc	909.2 ± 132.0	b	165.2 ± 7.9	a
	'Serrano'	1310.3 ± 50.6	cd	1338.8 ± 152.6	ab	245.1 ± 12.2	a
	'Jalapeño'	978.6 ± 34.0	d	1462.5 ±120.7	ab	274.3 ± 50.0	a
C. baccatum	'Aji Pineapple'	766.5 ± 83.6	a	1769.5 ± 42.2	a	261.3 ± 32.2	a
	'Bishops Crown'	1154.1 ± 100.2	ab	1397.5 ± 145.1	a	229.1 ± 38.0	a
	'Lemon Drop'	1000.0 ± 41.3	b	1580.7 ±166.7	a	233.5 ± 12.2	a
C. chinense × *C.frutescens*	'Bhut Jolokia'	2125.6 ± 110.7	/	3607.0 ± 333.8	/	467.8 ± 39.4	/

*a - e lower case letters in the same column denote statistical significant differences (α < 0.05) among different cultivars in the same species.

Table S5: Individual capsaicinoid content in pericarp (mg/100 g DW, mean ± SE) of different chili cultivars.

Species	Cultivar/capsaicinoids	Capsaicin		Dihydrocapsaicin		Nordihydrocapsaicin		Homocapsaicin		Homodihydrocapsaicin	
C. chinense	'Habanero Chocolate'	1172.2 ± 53.6	cd*	159.1 ± 10.0	cdef	25.6 ± 3.6	cd	19.0 ± 1.2	cd	9.0 ± 0.7	cde
	'Habanero Orange'	886.6 ± 21.4	de	145.8 ± 3.0	cdef	44.3 ± 0.4	bc	21.6 ± 0.5	bcd	19.3 ± 0.8	bcd
	'Habanero Yellow	914.7 ± 50.8	d	92.3 ± 5.8	ef	22.5 ± 4.1	cd	26.1 ± 2.1	bc	8.9 ± 1.5	cde
	'Aribibi Gusano'	1096.1 ± 108.5	d	122.9 ± 9.9	def	12.4 ± 0.9	d	15.8 ± 1.8	cde	5.5 ± 1.8	e
	'Moruga Scorpion yellow'	2061.9 ± 135.6	ab	278.1 ± 15.9	bcd	22.0 ± 1.8	cd	27.7 ± 1.3	bc	9.9 ± 0.1	cde
	'Naga Morich'	2080.3 ± 65.4	ab	337.6 ± 8.0	b	22.7 ± 1.1	cd	78.1 ± 4.5	a	20.3 ± 1.2	bc
	'7 Pot Primo Yellow'	809.3 ± 29.2	de	110.4 ± 17.2	ef	12.4 ± 2.1	d	8.2 ± 2.1	de	5.2 ± 1.0	e
	'Jay's Scorpion Peach'	2150.3 ± 11.4	ab	217.3 ± 5.8	bcde	10.9 ± 0.9	d	27.8 ± 1.3	bc	8.7 ± 0.6	de
	'Carolina Reaper'	2443.8 ± 59.2	a	644.4 ± 31.0	a	126.1 ± 7.8	a	37.2 ± 2.4	b	62.5 ± 1.1	a
	'Big Mustard Mama'	1535.3 ± 158.7	bcd	293.0 ± 13.1	bc	32.0 ± 2.9	bcd	11.0 ± 1.3	cde	7.6 ± 0.7	e
	'Borg 9 Pheno'	1943.4 ± 140.3	abc	333.5 ± 25.6	b	57.9 ± 6.8	b	38.2 ± 1.7	b	22.4 ± 7.0	b
	'Yellow Cap Mushroom'	112.6 ± 15.5	e	27.8 ± 4.6	f	4.6 ± 1.1	d	2.2 ± 0.4	e	1.5 ± 0.3	e
C. annuum	'Cayenne'	152.6 ± 14.7	a	42.5 ± 4.9	ab	29.7 ± 1.6	a	6.9 ± 0.5	a	12.9 ± 4.7	a
	'Bolivian Rainbow'	72.7 ± 4.6	b	16.7 ± 1.9	ab	6.7 ± 0.5	ab	1.0 ± 0.0	b	1.8 ± 0.2	b
	'Chilli AS- Rot'	52.6 ± 3.2	c	7.9 ± 0.6	b	3.8 ± 1.3	b	2.8 ± 0.1	ab	2.1 ± 0.6	b
	'Serrano'	116.7 ± 6.8	ab	54.3 ± 2.6	a	22.7 ± 2.0	ab	2.6 ± 0.2	ab	8.7 ± 1.8	ab
	'Jalapeño'	76.8 ± 12.8	b	21.2 ± 3.1	ab	17.8 ± 1.4	ab	2.5 ± 0.1	ab	5.5 ± 0.4	ab
C. baccatum	'Aji Pineapple'	246.7 ± 4.9	a	35.5 ± 1.4	a	8.3 ± 0.6	ab	14.0 ± 2.2	a	2.9 ± 0.2	ab
	'Bishops Crown'	38.6 ± 5.3	b	7.0 ± 0.1	b	1.9 ± 0.8	b	1.2 ± 0.3	b	0.3 ± 0.0	b
	'Lemon Drop'	194.9 ± 7.2	a	35.2 ± 1.3	a	8.8 ± 0.5	a	15.7 ± 3.8	a	3.1 ± 1.0	a
C. chinense × *C.frutescens*	'Bhut Jolokia'	1550.0 ± 70.2	/	170.4 ± 5.1	/	32.6 ± 0.6	/	38.3 ± 2.2	/	17.9 ± 2.3	/

*a to f lower case letters in the same column denote statistical significant differences ($\alpha < 0.05$) among different cultivars in the same species.

Table S6: Individual capsaicinoid content in placenta (mg/100 g DW, mean ± SE) of different chili cultivars.

Species	Cultivar/capsaicinoids	Capsaicin		Dihydrocapsaicin		Nordihydrocapsaicin		Homocapsaicin		Homodihydrocapsaicin	
C. chinense	'Habanero Chocolate'	2522.0 ± 124.7	a*	462.6 ± 33.2	ab	64.1 ± 2.2	cde	30.1 ± 2.5	d	23.7 ± 0.6	c
	'Habanero Orange'	2344.2 ± 74.7	a	580.3 ± 26.7	a	178 ± 8.6	a	54.7 ± 1.5	cd	82.1 ± 3.9	ab
	'Habanero Yellow	2334.3 ± 203.9	a	351.9 ± 29.3	bc	133.2 ± 16.2	ab	125.3 ± 5.8	ab	77.6 ± 4.4	ab
	'Aribibi Gusano'	2262 ± 129.2	a	226.1 ± 14.4	cde	60.0 ± 8.0	de	84.7 ± 8.3	bc	39.2 ± 3.5	c
	'Moruga Scorpion yellow'	2229.5 ± 72.8	a	195.8 ± 2.2	de	43.6 ± 2.1	de	44.6 ± 0.2	cd	25.2 ± 2.0	c
	'Naga Morich'	2364.1 ± 54.4	a	224.5 ± 15.1	cde	50.0 ± 6.9	de	156.8 ± 18.9	a	56.1 ± 7.9	bc
	'7 Pot Primo Yellow'	2049.6 ± 175.3	a	232.6 ± 11.6	cde	80.6 ± 9.4	bcde	40.1 ± 4.5	d	51.0 ± 5.0	bc
	'Jay's Scorpion Peach'	2315.3 ± 142.8	a	157.3 ± 14.7	e	37.9 ± 6.8	e	71.6 ± 6.7	cd	34.7 ± 4.4	c
	'Carolina Reaper'	2064.8 ± 165.7	a	301.4 ± 22.5	cd	160.6 ± 14.0	a	45.0 ± 4.0	cd	92.6 ± 8.2	a
	'Big Mustard Mama'	1994.4 ± 191.3	a	247.1 ± 27.1	cde	98.2 ± 4.4	bcd	39.9 ± 2.7	d	39.6 ± 1.3	c
	'Borg 9 Pheno'	1871.8 ± 105.3	a	268.0 ± 20.7	cde	121.6 ± 15.3	abc	67.4 ± 7.9	cd	57.6 ± 1.0	bc
	'Yellow Cap Mushroom'	830.7 ± 67.2	b	231.2 ± 21.4	cde	98.0 ± 7.2	bcd	34.1 ± 3.3	d	39.2 ± 3.9	c
C. annuum	'Cayenne'	306.2 ± 16.3	b	128.6 ± 4.2	b	126.6 ± 14.4	ab	25.8 ± 4.0	ab	55.7 ± 4.9	ab
	'Bolivian Rainbow'	573.3 ± 21.8	a	195.3 ± 9.6	a	102.3 ± 5.4	ab	10.2 ± 0.5	b	24.8 ± 1.3	ab
	'Chilli AS- Rot'	146.9 ± 8.1	b	33.5 ± 2.2	c	25.2 ± 4.5	b	7.8 ± 0.4	b	9.6 ± 0.3	b
	'Serrano'	237.9 ± 18.7	b	126.7 ± 4.3	b	46.9 ± 6.3	b	7.4 ± 2.6	b	20.2 ± 2.4	b
	'Jalapeño'	697.6 ± 59.8	a	212.9 ± 16.6	a	235.5 ± 9.3	a	42.2 ± 1.5	a	107.9 ± 4.8	a
C. baccatum	'Aji Pineapple'	840.6 ± 51.5	a	164.9 ± 8.8	a	104.1 ± 6.7	a	131.6 ± 1.7	a	42.8 ± 6.0	a
	'Bishops Crown'	448.3 ± 26.1	b	93.7 ± 15.7	b	47.4 ± 8.5	b	28.0 ± 1.4	b	15.2 ± 3.1	b
	'Lemon Drop'	863.3 ± 58.4	a	136.8 ± 13.9	ab	88.3 ± 5.3	ab	118.9 ± 13.8	a	45.1 ± 8.5	a
C. chinense × *C.frutescens*	'Bhut Jolokia'	2375.3 ± 119.3	/	273.4 ± 1.3	/	136.1 ± 0.6	/	128.7 ± 13.1	/	90.8 ± 4.2	/

*a to e lower case letters in the same column denote statistical significant differences (α < 0.05) among different cultivars in the same species.

Table S7: Individual capsaicinoid content in seeds (mg/100 g DW, mean ± SE) of different chili cultivars.

Species	Cultivar/capsaicinoids	Capsaicin		Dihydrocapsaicin		Nordihydrocapsaicin		Homocapsaicin		Homodihydrocapsaicin	
C. chinense	'Habanero Chocolate'	503.5 ± 35.2	abc*	48.6 ± 5.5	b	5.4 ± 0.5	c	3.8 ± 1.4	ab	1.6 ± 0.5	c
	'Habanero Orange'	727.7 ± 63.2	ab	101.4 ± 10.0	b	18.3 ± 1.8	abc	6.9 ± 1.1	ab	6.5 ± 0.7	abc
	'Habanero Yellow	618.4 ± 32.5	abc	58.4 ± 5.4	b	9.0 ± 0.4	abc	7.6 ± 2.0	ab	3.6 ± 1.2	bc
	'Aribibi Gusano'	788.6 ± 38.5	ab	111.9 ± 9.7	ab	10.2 ± 3.8	abc	12.8 ± 3.0	ab	4.5 ± 1.6	bc
	'Moruga Scorpion yellow'	324.4 ± 16.1	bc	47.0 ± 1.0	b	3.3 ± 0.1	c	2.7 ± 0.1	b	0.9 ± 0.1	c
	'Naga Morich'	644.5 ± 61.4	abc	112.6 ± 5.8	ab	7.3 ± 1.3	bc	20.7 ± 1.9	ab	4.6 ± 0.6	bc
	'7 Pot Primo Yellow'	401.9 ± 34.7	bc	87.5 ± 9.4	b	9.4 ± 1.2	abc	3.8 ± 0.5	ab	3.1 ± 0.4	bc
	'Jay's Scorpion Peach'	578.0 ± 31.6	abc	72.3 ± 2.9	b	7.7 ± 0.6	bc	6.8 ± 0.6	ab	3.0 ± 1.0	bc
	'Carolina Reaper'	1090.0 ± 96.3	a	204.8 ± 8.0	a	29.2 ± 7.8	a	19.0 ± 1.5	ab	12.9 ± 2.9	a
	'Big Mustard Mama'	894.8 ± 74.7	ab	97.5 ± 2.7	b	17.8 ± 6.6	abc	10.9 ± 1.4	ab	5.8 ± 0.3	abc
	'Borg 9 Pheno'	916.8 ± 43.4	ab	117.5 ± 5.9	ab	27.6 ± 7.4	ab	21.2 ± 1.5	a	10.8 ± 2.7	ab
	'Yellow Cap Mushroom'	79.8 ± 6.1	c	25.5 ± 3.0	b	6.8 ± 0.9	bc	2.8 ± 0.4	b	2.2 ± 0.2	c
C. annuum	'Cayenne'	43.1 ± 3.4	ab	13.2 ± 1.2	ab	11.3 ± 0.9	a	2.8 ± 0.3	a	5.6 ± 0.3	a
	'Bolivian Rainbow'	30.8 ± 1.1	b	9.5 ± 1.3	ab	4.4 ± 0.7	a	0.5 ± 0.0	a	0.9 ± 0.1	a
	'Chilli AS- Rot'	20.3 ± 1.9	b	3.0 ± 0.2	b	1.5 ± 0.5	a	1.1 ± 0.5	a	0.6 ± 0.2	a
	'Serrano'	38.8 ± 2.1	ab	21.5 ± 3.5	ab	5.8 ± 1.4	a	0.9 ± 0.4	a	2.4 ± 0.5	a
	'Jalapeño'	107.6 ± 7.6	a	29.0 ± 2.4	a	16.8 ± 8.6	a	2.9 ± 1.5	a	5.0 ± 2.8	a
C. baccatum	'Aji Pineapple'	182.7 ± 10.5	a	43.2 ± 7.0	a	8.4 ± 1.9	a	10.4 ± 1.4	a	1.8 ± 0.2	a
	'Bishops Crown'	48.5 ± 7.1	b	12.5 ± 1.3	b	1.7 ± 0.1	a	1.1 ± 0.2	b	0.3 ± 0.0	a
	'Lemon Drop'	162 ± 10.9	ab	33.9 ± 1.8	a	5.6 ± 2.3	a	8.4 ± 2.9	ab	1.7 ± 0.8	a
C. chinense × *C.frutescens*	'Bhut Jolokia'	1011.4 ± 74.4	/	92.1 ± 6.9	/	14.4 ± 2.2	/	12.3 ± 2.1	/	5.2 ± 0.3	/

*a to c lower case letters in the same column denote statistical significant differences ($\alpha < 0.05$) among different cultivars in the same species.
